# An Assessment of Quaternary Structure Functionality in Homomer Protein Complexes

**DOI:** 10.1093/molbev/msad070

**Published:** 2023-03-22

**Authors:** György Abrusán, Carles Foguet

**Affiliations:** Department of Public Health and Primary Care, School of Medicine, University of Cambridge, Cambridge, United Kingdom; Department of Public Health and Primary Care, School of Medicine, University of Cambridge, Cambridge, United Kingdom

**Keywords:** protein complexes, homomers, neutral evolution, ligand binding, coevolution

## Abstract

It has been recently suggested that a significant fraction of homomer protein–protein interfaces evolve neutrally, without contributing to function, due to a hydrophobic bias in missense mutations. However, the fraction of such gratuitous complexes is currently unknown. Here, we quantified the fraction of homodimers where multimerization is unlikely to contribute to their biochemical function. We show that: 1) ligand binding-site structure predicts whether a homomer is functional or not; the vast majority of homodimers with multichain binding-sites (MBS) are likely to be functional, while in homodimers with single-chain binding-sites (SBS) and small to medium interfaces, quaternary structure is unlikely to be functional in a significant fraction—35%, even up to 42%—of complexes; 2) the hydrophobicity of interfaces changes little with the strength of selection, and the amino acid composition of interfaces is shaped by the “hydrophobic ratchet” in both types, but they are not in a strict equilibrium with mutations; particularly cysteines are much more abundant in mutations than in interfaces or surfaces; 3) in MBS homomers, the interfaces are conserved, while in a high fraction of SBS homomers, the interface is not more conserved than the solvent-accessible surface; and 4) MBS homomer interfaces coevolve more strongly with ligand binding sites than the interfaces of SBS homomers, and MBS complexes have higher capacity to transfer information from ligands across the interfaces than SBS homomers, explaining the enrichment of allostery in the former.

## Introduction

Most proteins form protein complexes to perform their functions, which can be divided into two large categories: homomers and heteromers (Marsh and Teichmann 2015). Heteromers are complexes formed by different proteins, while homomers are complexes formed by multiple units of the same protein. Among the biounits of the 170 k protein data bank entries, homomers outnumber heteromers 2.5 times and are the most abundant protein complex type, particularly in prokaryotes. Despite being very common, the degree to which the quaternary structure of homomers contributes to their biological function remains an open question. In many cases, multimerization is known to be relevant: mutations in protein interfaces are frequently pathogenic (Yates and Sternberg 2013; Sahni et al. 2015; Livesey and Marsh 2022); the interfaces of homomers are frequently conserved across long evolutionary distances (Levy et al. 2008); and the importance of quaternary structure for function has been validated for a large number of proteins (see reviews by Marianayagam et al. (2004), Perica et al. (2012), and Marsh and Teichmann (2015)). Some homomers are known to exist in an equilibrium with their monomeric form (Nooren and Thornton 2003; Dey et al. 2010; Acuner Ozbabacan et al. 2011), and recent experimental work indicates that a mixture of different forms is the norm in solvent, rather than the exception (Marciano et al. 2022). Such “weak” complexes are nevertheless considered functional, and important in allostery, signaling, or regulation.

Most genomic traits, like genome size or the amount of non-coding sequence, scale with effective population size and strength of selection in an organism (Lynch 2007). However, homomers do not show this trend, and in many species, the same functions are performed by homomers which are homologous, but have different topologies or unit numbers (Lynch 2013). This was interpreted as a sign of neutrality, that is, that quaternary structure may not contribute to biological function in many homomers (Lynch 2013; Hagner et al. 2018). This is supported by theoretical findings suggesting that structural similarity enhances the formation of protein interactions (Lukatsky et al. 2007), that homomers might be a priori biased towards being symmetric, even before selection can act on them (André et al. 2008), and that some binding interactions with ligands or other proteins might be evolutionary “spandrels” that stabilize protein folds, without providing fitness advantage (Manhart and Morozov 2015).

**Fig. 1.**
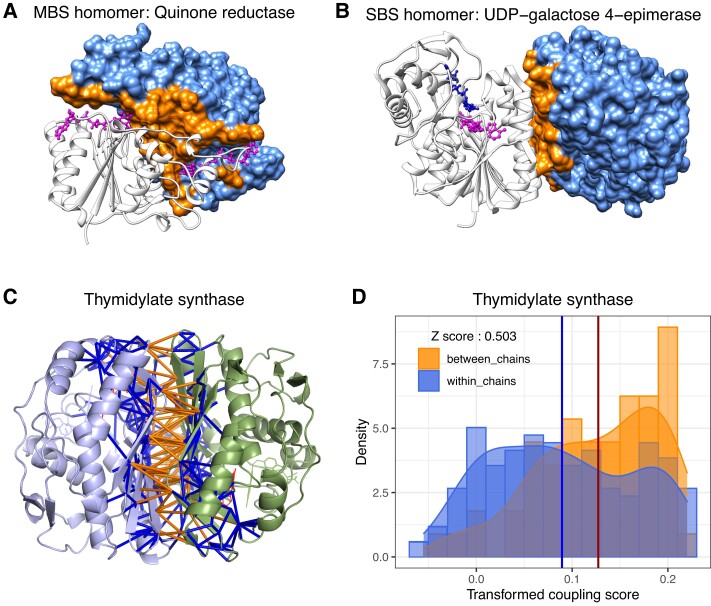
(*A*) MBS homomer: human quinone reductase type 2 (PDB id: 1QR2), with two flavin-adenine dinucleotide ligands (magenta) between the two chains. (*B*) SBS homomer: human UDP-galactose 4-epimerase (PDB id: 1EK6), with ligands binding only one chain. On both structures (*A* and *B*), surface residues of chain B are colored light blue, while interface residues of chain B are orange. (*C*) Interactions of interface residues of the *Escherichia coli* Thymidylate synthase (PDB id: 1TLC), an MBS homomer. Orange rods represent interactions across the interface; and blue rods indicate interactions of interface residues with non-interface residues. (*D*) The corresponding distributions of evolutionary coupling scores, in the same color-coding. The blue vertical line represents the mean coupling score of the interface with non-interface interactions (blue), and the red vertical line represents the mean score of interactions across the interface (orange). The *Z*-score was calculated as the difference between the two means, divided by the SD of the blue distribution. Both distributions were transformed with the Yeo–Johnson transformation (a generalized Box–Cox transformation); and the lambda parameter was chosen to maximize the normality of the blue (interface—non-interface residues) distribution.

Subsequently, using ligand binding ability as the proxy of function, we have examined the contribution of quaternary structure to the evolution of function in protein complexes and monomers (Abrusán and Marsh 2018; Abrusán and Marsh 2019a). Homomers where binding sites are formed by multiple protein chains (“multichain binding sites”, MBS homomers, fig. 1*A*) show much higher conservation of binding sites and quaternary structure than homomers where the binding sites are restricted to a single chain (“singlechain binding sites”, SBS homomers, fig. 1*B*) or monomers (Abrusán and Marsh 2018). The similarity of the evolution of ligand binding in SBS homomers and monomers was interpreted as an indication that in SBS homomers, quaternary structure frequently does not influence the biochemical function, and its neutral evolution is likely to be frequent (Abrusán and Marsh 2018). In addition, the characteristics of the folds of SBS homomers are very similar to monomers, also suggesting that in these complexes, multimerization frequently does not contribute to function (Abrusán and Marsh 2019a). Recently, Hochberg et al. have provided experimental evidence that in steroid receptors, dimerization does not have a detectable contribution to their biochemical function (Hochberg et al. 2020; Schulz et al. 2022). This was attributed to constructive neutral evolution: in natural genetic variation, the high frequency of missense mutations that create hydrophobic amino acids can result in the formation of hydrophobic patches on the surface, which may become protein–protein interfaces, because protein–protein interfaces are typically much more hydrophobic than solvent accessible surface. Such interfaces may persist for long evolutionary periods, even if they do not contribute to function, because once they were formed, exposing them to solvent can be deleterious, and purifying selection can maintain them (Hochberg et al. 2020). However, currently it is unclear whether this “hydrophobic ratchet” is a major force driving the evolution of protein complexes.

In this paper, using homodimers, we examined what is the fraction of homomers where multimerization is unlikely to contribute to their biochemical function, and to what degree the hydrophobic ratchet shapes the amino acid composition of interfaces. To test this, we examined whether 1) the composition of interfaces is in equilibrium with the missense mutations that occur in them; 2) whether their interfaces are conserved; 3) whether interface and ligand-binding residues coevolve; and 4) whether there is information transfer across the interface of different homodimers. We found clear signs that the hydrophobic ratchet shapes the composition of interfaces, however, at least in humans, interface composition is not in a strict equilibrium with mutations, and such equilibrium may not even be possible. The analysis of interface conservation indicates that in a significant fraction of homodimers with single-chain binding sites, the interface is not more conserved than the solvent accessible surface. Coevolution between residues has been used to analyze protein function for more than two decades (Lockless and Ranganathan 1999; Süel et al. 2003), but in recent years, methods based on correlated evolution of residues (in combination with machine learning) have revolutionized protein structure prediction (Humphreys et al. 2021; Jumper et al. 2021), and has also been successfully used to identify protein–protein interfaces (Hopf et al. 2014; Ovchinnikov et al. 2014; Humphreys et al. 2021), or pathogenic mutations (Hopf et al. 2017; Frazer et al. 2021). Our analyses indicate that the type of binding site (MBS vs. SBS) is highly indicative whether multimerization contributes to function: in MBS homomers, we detected strong coevolution between interface residues and ligand-binding residues, while in SBS homomers, it is much weaker. Additionally, by analyzing the motions of residues, we show that in MBS homomers, interface residues that coevolve with ligand binding residues can also transfer information between the subunits of a complex, while in SBS homomers, the capacity for such information transfer is much weaker. The comparison of different methods used for predicting functionality indicates that when the interface is smaller than 100 residues, the fraction of gratuitous SBS homomers is likely to be close to 35%, and can be as high as 42%. Finally, using human genome-scale metabolic networks, we also show that these structural differences translate to functional differences, and the loss of MBS proteins is more likely to result in blocked reactions than the loss of SBS homomers. This suggests that the reactions catalyzed by MBS homomers are less buffered by the topology of the network, and are potentially regulated differently than the ones catalyzed by SBS homomers.

## Results

### Datasets

We compiled two datasets of homodimers for the downstream analyses; one based on the entire protein data bank (PDB), which includes both prokaryotes and eukaryotes (“All”, 992 proteins), and a much smaller one (148 proteins), using only the human entries in the PDB (supplementary tables S1 and S2, Supplementary Material online). Both datasets contain only entries that bind ligands, and are either MBS or SBS homomers. Structures containing only metal ligands were not included. In the first, full dataset the sequences were filtered for redundancies at a 30% similarity level, and were required to have a minimum 90% overlap between their structure and UniProt sequence. Proteins in the human dataset were not filtered for redundancies, and we required a lower 75% overlap between the structures and their UniProt sequence (see Methods). While the human dataset is of lower quality than the full set, it allows comparisons with other resources like genetic variants, or metabolic networks.

### Residues Coevolve in Most Interfaces

We examined whether interfaces are under similar evolutionary constraints in MBS and SBS homomers by testing whether residue–residue interactions across the interface coevolve more strongly than the interactions between interface and non-interface residues (fig. 1*C* and *D*). This approach has the advantage that it might identify crystal artifacts even between real interfaces. For example, a protein may have an interface, which normally forms a heteromer with a different protein. A homodimer of such proteins in the PDB might be a crystallographic artifact, even if the interface residues are conserved. However, such crystallographic homodimers are not expected to have coevolving interface residues.

The strength of coevolution for every possible residue pair in the protein sequences was estimated with the EVcouplings tool (Hopf et al. 2019) (Methods), which measures coevolution between residue pairs across homologs. Using the distributions of the coupling scores in these two sets of residue pairs, we calculated a *Z*-score as the difference between their means divided by the standard deviation of the interface/non-interface distribution (see fig. 1*C* and *D*; in this analysis, the coupling score distributions were transformed to improve their normality [see Methods], however, the results are similar with non-transformed data). Positive *Z*-scores indicate stronger coevolution between interface residues than between interface and non-interface residues and are a signature of strong selection on interfaces (note that values smaller than zero do not necessarily imply the complete absence of coevolution between interface residues).

**Fig. 2.**
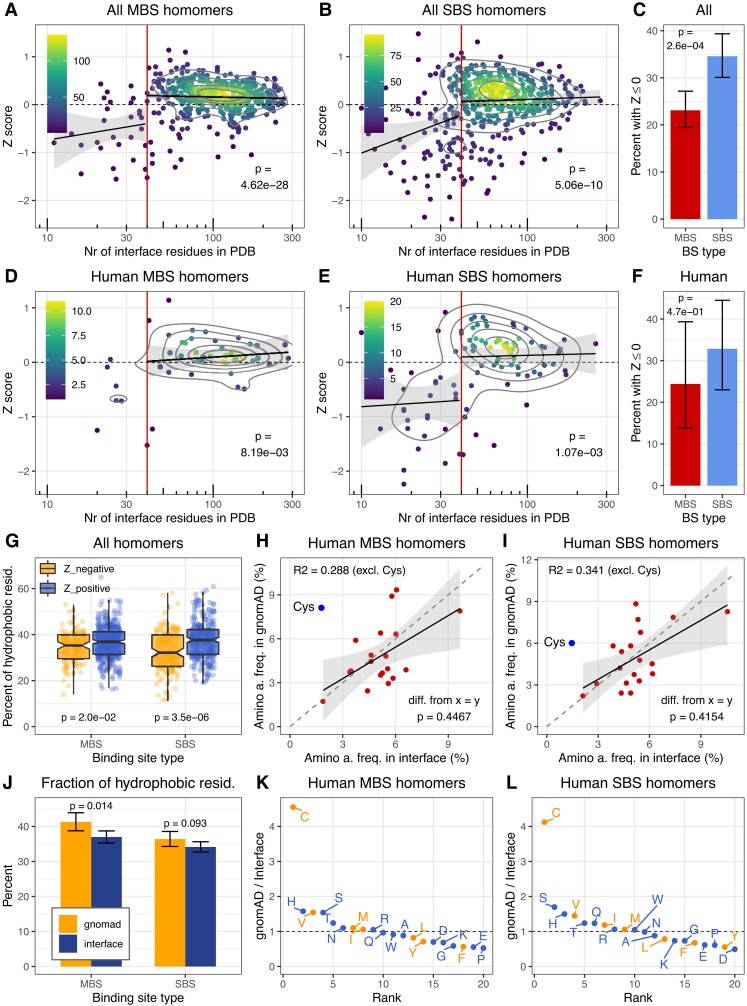
Coevolution of residues in interfaces and their amino acid frequencies. (*A* and *B*) In the full dataset, in both types of protein complexes, interface interactions coevolve stronger than the interactions between interface and non-interface residues (*Z* > 0; Wilcoxon tests, see also supplementary fig. S1, Supplementary Material online), indicating that the interactions within the interface are under strong selection in most dimers. This trend disappears only, when the total number of interface residues falls below 40 (which corresponds to an interface area of ∼1000 Å^2^), suggesting that most interfaces below this threshold might not be real or stable in the homologs, and such complexes were excluded from the downstream analyses. The intensity of yellow indicates the density of the points and *P*-values indicate the significance of the difference from 0. (*C*) The fraction of proteins with zero or negative *Z* is significantly higher in SBS homomers than in MBS homomers (test of proportions). (*D*, *E*, and *F*) The human dataset shows qualitatively similar characteristics as the full, nonredundant dataset. (*G*) The fraction of hydrophobic residues in the interfaces of complexes with negative *Z* is somewhat lower than in complexes with positive *Z*, however, they are still much more hydrophobic than the 10–20% expected in surfaces exposed to solvent. (*H* and *I*) The frequency of specific amino acids in the interface and gnomAD missense mutations (excluding cysteine) shows a similar, positive correlation in both homomer types, with a slope that is not significantly different from one (*F* statistic). (*J*) The frequency of hydrophobic amino acids is just minimally or not higher in gnomAD missense mutations than in protein interfaces (tests of proportion). (*K* and *L*) The frequency of cysteines in gnomAD missense mutations is ∼4 times higher than in the (already quite hydrophobic) interfaces, indicating strong selection against cysteines, even in interfaces.

The results indicate that *Z*-score is positive in most homomers, and interactions across interfaces coevolve more strongly than the interactions between interface and non-interface residues (fig. 2*A*–*F*). The full nonredundant (fig. 2*A*–*B*) and human sets (fig. 2*D*–*E*) are qualitatively similar, and show that *Z* depends on the size of the interface: when the total number of interface residues is below 40 in a complex, *Z*-score drops sharply, suggesting that below this threshold, even in biounits, many homodimers might be crystallographic errors, rather than stable complexes, or their interfaces are highly unstable across evolution. Interfaces with 40 contacting residues correspond to an interface area of ∼1044 Å^2^ (using the DSSP tool), which is in good agreement with comparisons of crystallographic and biological interfaces (Baskaran et al. 2014) that show that the vast majority of crystallographic interfaces are smaller than ∼1000 Å^2^. In addition, in heteromers, interface residues were shown to coevolve much less in transient than obligate complexes (Mintseris and Weng 2005), suggesting that besides crystal artifacts, the drop in *Z*-scores in our dataset might also reflect major differences in the stability of the interface. Therefore, we excluded homomers with less than 40 interface residues from all downstream analyses. The number of complexes with zero or negative *Z* is significantly different in the two complex types (interface size 40+, fig. 2*C* and *F*): 23% of MBS homomers have zero or negative *Z* while 34.5% of SBS homomers. Interfaces with negative *Z* have fewer hydrophobic residues (defined as the following amino acids: C, F, I, L, M, V, Y) than interfaces with positive *Z*, particularly in SBS homomers (fig. 2*G*, see also supplementary fig. S1, Supplementary Material online); however, even in these interfaces, their frequency is much higher than the 10–20% that characterizes solvent accessible surfaces.

Taken together, these results indicate that the majority of interfaces in our dataset are maintained by selection, both in MBS and SBS homomers. However, this does not necessarily mean that they do contribute to biological function because even if interfaces are formed due to a neutral process, once they exist, exposing them to solvent is deleterious. Selection may simply operate on assembly and ensure that the interfaces fit tightly, and no large hydrophobic patches are exposed to solvent (Levy et al. 2012).

### The Amino Acid Composition of Missense Mutations and Interfaces is Correlated

Next, we asked whether there is an equilibrium between the amino-acid frequencies of the interfaces and the missense mutations in them, that is, how similar is their amino-acid composition. We hypothesized that if the hydrophobic ratchet is a significant factor determining the evolution of interfaces, then the amino acid composition of interfaces and new amino acids created by missense mutations will converge over time. Using the human homodimer set and the genome aggregation database (gnomAD) (Karczewski et al. 2020), we examined whether the composition of the interface and solvent-accessible surface is correlated with the composition of the missense mutations in them (see Methods). Missense mutations in gnomAD are only moderately filtered by selection: 84.2% and 88.1% of expected mutations are observed in MBS and SBS transcripts; thus, we expected that their amino acid composition is not dramatically different from neutral mutations. Altogether, we identified 1392 missense mutations in the interfaces of MBS homomers, and 1948 in SBS homomers. We found significant positive correlations between interface and gnomAD amino acid frequencies with a slope that is not significantly different from one (*F* statistic; fig. 2*H* and *I*, excluding cysteine) in both complex types, and the overall frequency of hydrophobic residues (C, F, I, L, M, V, Y) is also similar (fig. 2*J*). In the case of solvent-accessible surface (3295 missense mutations in MBS and 7617 in SBS complexes), the slope of the correlation between gnomAD and surface residue frequencies is significantly different from one (supplementary fig. S2*A*and*B*, Supplementary Material online), indicating that the hydrophobic ratchet does shape the interfaces. However, the correlations are weak (fig. 2*H*–*I*), and the two complex types (MBS vs. SBS) do not differ substantially. Additionally, if leucine, the amino acid with the highest frequency in the surface is removed, then the interface-correlations (fig. 2*H* and *I*) are not or just marginally significant (although their slope remains largely unchanged).

Of all amino acids, cysteine stands out, as it has approximately four times higher frequency among gnomAD missense mutations than among interface residues (fig. 2*K* and *L*), primarily due to CpG mutations [C/T] in arginine codons, and A/G transitions in tyrosine codons. In solvent-accessible surfaces, its frequency is 6- to 9-fold higher in gnomAD than in the protein (supplementary fig. S2*D*and*E*, Supplementary Material online). While gnomAD variants are generally not thought to be pathogenic, this nevertheless suggests that there is strong selection against cysteines in interfaces and surfaces, most likely due to their reactivity, and ability to form disulphide bridges, which may result in deleterious cross-linking between proteins (Iqbal et al. 2020). Overall, our findings indicate that the hydrophobic ratchet does shape the amino acid composition of interfaces, however, they are not in a strict equilibrium with missense mutations. Due to biological constraints, for certain amino acids like cysteine reaching equilibrium frequencies may not even be possible, and it is also likely that maintaining correct assembly creates selective pressure that pushes interfaces away from equilibrium frequencies.

### Conservation of Interfaces

**Fig. 3.**
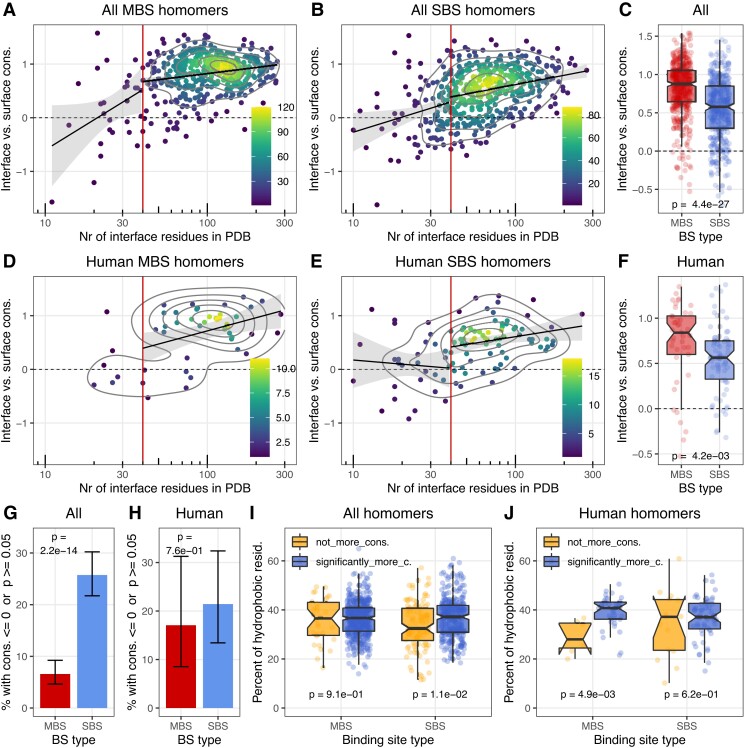
Conservation of interfaces in homodimers. Conservation was calculated within each structure as the difference between the ConSurf scores of surface and interface residues. Positive values indicate that the interface is more conserved than the surface. (*A* and *B*) Interface conservation vs. the size of the interface in the full dataset. In both dimer types, the majority of interfaces are more conserved than the surface. (*C*) Interfaces of MBS homomers are significantly more conserved than the interfaces of SBS homomers. (*D*–*F*) The human dataset shows a similar pattern to the full set. (*G* and *H*) In the full dataset the fraction of complexes where interface is not significantly more conserved than the surface is much higher in SBS homomers than in MBS homomers, but the difference is not significant in the human set. (*I* and *J*) The fraction of hydrophobic residues in interfaces that are significantly more conserved than the surface, and in the ones that are not. The level of interface hydrophobicity shows a qualitatively similar pattern to figure 2*G*, indicating that high interface conservation is not necessary for the evolution of hydrophobic interfaces (except for panels *A*–*B* and *D*–*E*, only structures with 40+ interface residues were used).

In addition to residue coevolution, we also examined whether interfaces are conserved in the two homomer types. We used the conservation scores of the ConSurf database (Ben Chorin et al. 2020) to calculate the difference between the interface and solvent-accessible surface for each structure (see Methods). Several studies have reported that interfaces are somewhat more conserved than the solvent accessible surface (Valdar and Thornton 2001; Mintseris and Weng 2005; Jack et al. 2016), and our results corroborate this: in most structures, the interface is significantly more conserved than the surface, both in the full and the human dataset (fig. 3*A*–*B* and *D*–*E*). However, the difference is much more pronounced in MBS homomers (fig. 3*C* and *F*), and in the full dataset, it remains highly significant even if interface size is included as a covariate with *P* = 5.9e^−15^ for the full dataset and *P* = 0.27 for the human dataset in an analysis of covariance (ANCOVA). Also, in the full dataset, the fraction of interfaces that are not more conserved than the surface is much higher in SBS homomers (fig. 3*H*). The difference is partly caused by the closeness of the MBS ligands to the interface, which, besides influencing the conservation of the interface residues that interact with the ligand, due to long range effects (Jack et al. 2016), is likely to influence a significant fraction, if not most of the interface.

The comparison of the hydrophobicity of interfaces that are significantly more conserved than the surface and the ones that are not more conserved shows that the two are not dramatically different (fig. 3*I* and *J*), and the pattern is comparable to coevolution across the interfaces (fig. 2*G*). This indicates that the degree of conservation has little effect on hydrophobicity, which is in agreement with the idea that the main process that drives the hydrophobicity of interfaces is not selection, but a neutral process (i.e., the hydrophobic ratchet). However, it also means that the ratchet shapes both functional and non-functional interfaces, and the hydrophobicity of an interface predicts poorly whether an interface is neutral, adaptive, or at least conserved. The hydrophobic ratchet is likely to influence also the evolution of other types of hydrophobic regions in proteins not just interfaces, such as membrane-bound regions. Thus, we also considered the possibility that some of the interfaces with low conservation might actually be membrane-bound regions forming real or crystallographic interfaces (see PDB id 4FMM for an interesting case, where a membrane-bound region also forms an interface), but the relatively few membrane proteins in our dataset do not show a qualitatively different pattern from the rest (data not shown).

### Ligand-Binding Residues Show Strong Coevolution with Interface Residues in MBS Homomers but not SBS Homomers

**Fig. 4.**
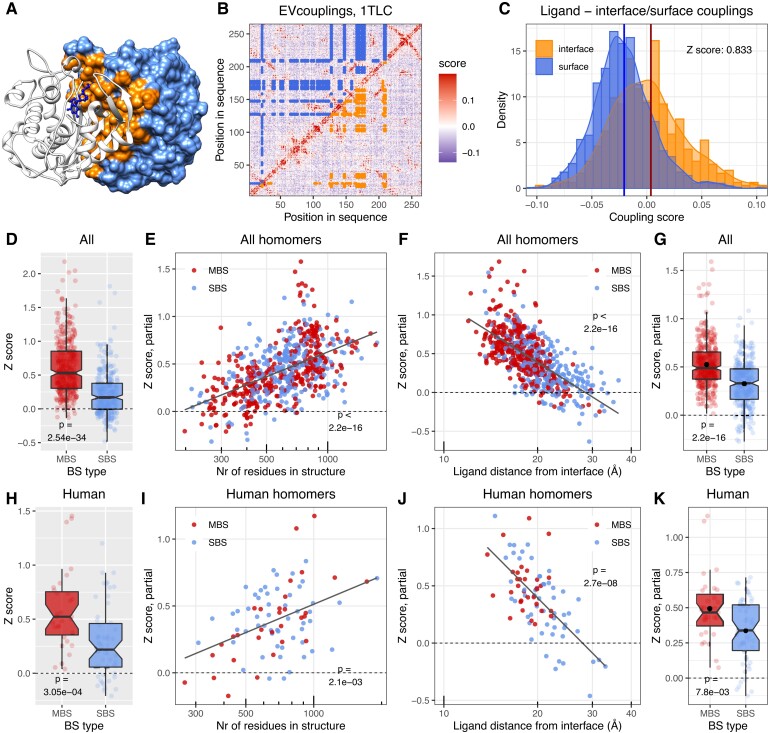
In MBS homomers ligand-binding residues coevolve much stronger with the interface than in SBS homomers. (*A*) The structure of *E*. *coli* Thymidylate synthase, an MBS homomer. The interface on chain B is highlighted with orange and the solvent-accessible surface with light blue. (*B*) Matrix of evolutionary coupling scores in Thymidylate synthase. In the upper triangle, blue points highlight the couplings between 2'-deoxyguanosine-5'-monophosphate (DGP)-binding and surface residues; in the lower triangle, orange indicates the couplings between DGP-binding and interface residues. (*C*) The distributions of ligand-surface couplings (blue) and ligand-interface couplings (orange) in Thymidylate synthase. The coupling between the ligands and interface residues was measured as *Z*-score. It was calculated as the difference of the interface distribution's mean from the surface distribution's mean (vertical lines), divided by the SD of the surface (blue) distribution. (*D*) The raw *Z*-scores of MBS and SBS homodimers in the full dataset show a highly significant difference between the two (Wilcoxon test). (*E*, *F*, and *G*) ANCOVA partial correlations between *Z*-score and the size of the structure (*E*), distance of ligands from the interface in Å (*F*), and binding site type (*G*). While the strength of coupling between the interface and ligand (*Z*-score) depends on other factors, when the effect of covariates is removed, binding site type still remains a highly significant factor. See the full ANCOVA results in supplementary table S3, Supplementary Material online and also supplementary figure S4, Supplementary Material online. (*H*, *I*, *J*, and *K*) The human dataset shows a similar trend as the full dataset (see supplementary table S3, Supplementary Material online for the ANCOVA results and supplementary fig. S4, Supplementary Material online). The black dots on panels *G* and *K* indicate the mean.

To test whether interfaces are necessary for performing the biochemical function of the proteins, we next examined whether interfaces coevolve with ligand-binding residues (LBRs). Multimerization may modulate function in several ways; for example, binding sites may be active only in complex (Bern and Tobi 2022), interfaces may influence their specificity (Rausell et al. 2010), or the ligands may communicate allosterically across the interface (Stefan and Le Novère 2013). We expected that in many complexes where multimerization does contribute to function, LBRs coevolve more strongly with interface residues than with the solvent-accessible residues of the surface. To test this, we calculated a *Z*-score using the evolutionary coupling scores between LBRs and solvent-accessible surface residues, vs. LBRs and interface residues (fig. 4*A*–*B*). *Z* was calculated as the difference between the means of the two distributions, divided by the standard deviation (SD) of the LBR-surface coupling distribution (fig. 4*C*). The distributions of coupling scores were not transformed. Metals were not used, and when multiple ligands were present in a structure, the ligand with the largest *Z*-score was selected. In MBS homomers, only MBS ligands were used, even if SBS ligands were also present in the structure.

The majority of MBS dimers have two symmetrical multichain ligand-binding sites, however, a minority has only one binding site. Such complexes are also likely to function differently (i.e., are involved in signaling or regulation rather than in enzymatic activity); thus, we performed separate analyses to compare them with SBS homomers. In the case of MBS homomers with two multichain binding sites, we found that in MBS homomers, LBRs and interface residues coevolve more strongly than in SBS homomers, both in the full, nonredundant and human datasets (fig. 4*D* and *H*). Since the topologies of the two complex types are different, for example in MBS homomers, ligands are located closer to interface residues than in SBS homomers, and MBS homomers have larger interfaces, we examined whether the effect of binding-site type on interfaces (*Z*) remains significant if several additional covariates are added to the analysis. In addition, as the coupling scores of EVcouplings are based on the properties of an alignment, and not individual sequences, differences in the conservation/coevolution of ligand binding residues may also affect the results. To control for this, we also included the rate of coevolution between ligand-binding residues in the analyses as a covariate. The magnitude of coevolution between LBRs was also estimated with a *Z*-score, where the mean of couplings between LBRs was compared to the mean and standard deviation of couplings of LBRs with surface residues. We performed ANCOVA with the following, log-transformed covariates in addition to binding site type: the size of the structure (residues), interface size (residues), ligand distance from the interface (Å), coevolution between ligand binding residues, and the effective number of sequences of the alignments. The effects of interface size and effective number of sequences were not significant, and they were subsequently removed with a backwards elimination procedure. The results show that even though other variables, particularly the size of the structure and ligand distance from interfaces, clearly influence the strength of coevolution between LBRs and interface (fig. 4*E* and *F*), the effect of binding site type remains highly significant (fig. 4*G*, see also supplementary table S3, Supplementary Material online for the full ANCOVA table). The much smaller human dataset generally shows a similar pattern to the full set (fig. 4*H*–*K*, see supplementary table S3, Supplementary Material online for the full ANCOVA table). In the case of MBS homomers with a single multichain binding site (supplementary fig. S3, Supplementary Material online and supplementary table S4, Supplementary Material online), we observed similar patterns, both in the full and human datasets, although in the human set the number of such complexes is very small. To maintain consistency, on supplementary figure S3, Supplementary Material online, we fitted the same complex model as on figure 4, even when the effect of a covariate was not significant (e.g., the size of the structure in the human set). Taken together, these results indicate that in most MBS homomers, multimerization does contribute to function, while multimerization is likely to be unnecessary in many SBS homomers.

### Coevolving Residues Display Correlated Motions in MBS Homomers

While interface residues show clear coevolution with ligand-binding residues in some complexes, the contribution of individual residues to this pattern is variable. It has been shown that the evolutionary characteristics and dynamical properties of protein residues are correlated, and residues that are dynamically coupled are also frequently functionally coupled (Liu and Bahar 2012; Campitelli et al. 2020). Recently, Sapienza et al. (2019) have demonstrated that in Thymidylate synthase, an allosteric MBS homomer that is a major chemotherapeutic target in cancer (Wilson et al. 2014), ligands communicate across the interface, and a small number of “hot-spot” residues are particularly important in transmitting motions between the subunits (Sapienza et al. 2019). We found that these hot-spot residues also coevolve more strongly with ligand binding residues than other interface residues (supplementary fig. S4, Supplementary Material online); thus, we examined whether there is a general association between the strength of coevolution and correlated motions between ligand binding and interface residues.

**Fig. 5.**
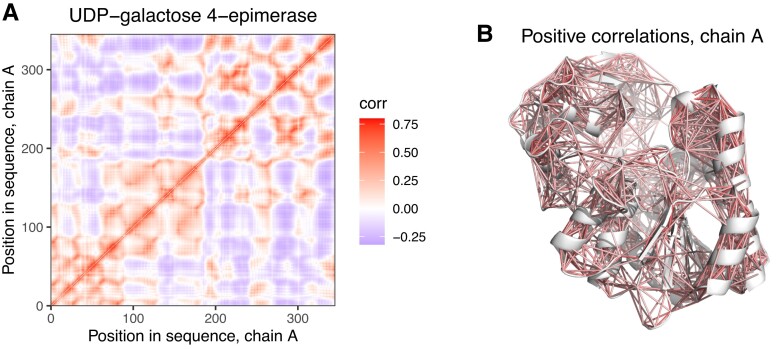
Correlated motions identified by normal mode analysis (NMA) (*A*) Dynamic cross-correlation matrix of residues on chain A of UDP-galactose 4-epimerase (see fig. 1*B* for the dimer), made with all-atom NMA, using all modes. (*B*) The location of residue pairs with positively correlated motions (*R* > 0.2) on chain A of the dimer (fig. 1*B*).

**Fig. 6.**
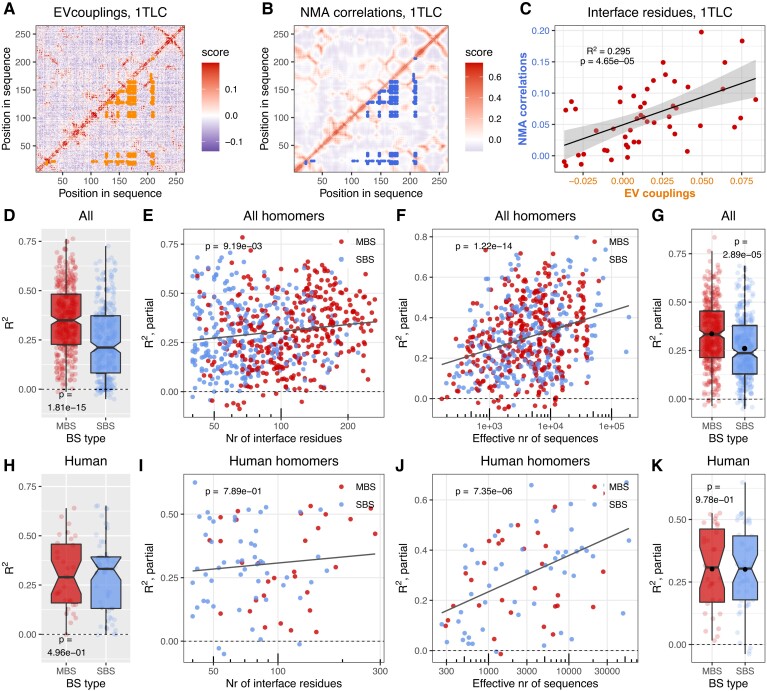
The coevolution and dynamics of ligand and interface residues are correlated. (*A*) Matrix of evolutionary couplings in *E*. *coli* Thymidylate synthase (PDB id: 1TLC). Orange points indicate couplings between ligands binding residues and interface residues. (*B*) Correlated motions in chain A of the Thymidylate synthase dimer. Blue points indicate the correlated motions between ligand-binding and interface residues. (*C*) Correlation between EVcouplings and NMA. Each point represents an interface residue, for which the x coordinate is its average coupling with the ligand-binding residues (see panel *A*), while its y coordinate is the average dynamic correlation in the NMA cross-correlation matrix (see panel *B*) with the ligand-binding residues. (*D*) The *R*^2^ values in the full dataset indicate a highly significant difference between MBS and SBS homomers (Wilcoxon test). (*E*, *F*, and *G*) ANCOVA partial correlations between *R*^2^ and the number of interface residues (*E*), effective number of sequences (*F*), and binding site type (*G*). The effect of binding site type remains significant, when the effects of covariates are removed. See the full ANCOVA results in supplementary table S4, Supplementary Material online and also supplementary figure S6, Supplementary Material online. (*H*, *I*, *J*, and *K*) In the human dataset, we found no clear effect of the type of binding site on *R*^2^. See also supplementary table S4, Supplementary Material online and supplementary figure S6, Supplementary Material online. The black dots on panels *G* and *K* indicate the mean.

To test this, we performed all-atom normal mode analysis (NMA) on all homodimer structures with the Bio3D R package (Grant et al. 2021), and determined matrices of cross-correlated motions between all residue pairs of the structures (see Methods and fig. 5). In our benchmarks, we found that within a subunit of a dimer, the correlation (*R*^2^) between matrices obtained with NMA and with molecular dynamics is high, usually above 0.8 (data not shown). Next, for each interface residue in the structures, we calculated its average coupling score and average correlation in the NMA cross-correlation matrix with the ligand-binding residues, and calculated the Pearson coefficient of determination (*R*^2^) between the two (see fig. 6*A* for couplings scores between the interface and ligands in Thymidylate synthase, fig. 6*B* for cross-correlated motions between them, and fig. 6*C* for the correlation between the two). Similar to the previous analysis (fig. 4), when multiple ligands were present in a structure, the ligand with the largest *R*^2^ was selected, and metals were excluded. In MBS homomers, only MBS ligands were used, even if SBS ligands were present in them, and MBS homomers with two or a single multichain ligand were analyzed separately (fig. 6 and supplementary fig. S5, Supplementary Material online).

For both MBS homomer types, we found a similar pattern; in the full dataset in MBS homomers, there is a much stronger association between coevolution and correlated motions than in SBS homomers (fig. 6*D* and supplementary fig. S5*A*, Supplementary Material online), while in the human dataset, there is no significant difference (fig. 6*H* and supplementary fig. S5*E*, Supplementary Material online). We also performed an ANCOVA using the same covariates as above (the size of the structure and the coevolution of LBRs were removed with a backwards elimination procedure due to their non-significant effect). Compared to the analysis of ligand-interface coevolution (fig. 4), the covariates have a much weaker impact on *R*^2^ (fig. 6*E*, *F*, *I*, and *J*), but their inclusion does not remove the effect of binding site type (fig. 6*G*, see also supplementary table S5 and table S6, Supplementary Material online for full ANCOVA results). Taken together, these findings indicate that many MBS homomers have the capacity to transmit information from ligands across their interface and explain why allostery is much more common among them than in SBS homomers (Abrusán and Marsh 2019b).

Next, we examined which residues contribute the most to the observed correlations between motions and coevolution. In MBS homomers, ligands that bind both protein subunits are typically closer to interfaces than ligands in SBS homomers (see fig. 4*F* and *J*). While the average distance of ligands from the interface has only a minor effect on *R*^2^ (supplementary table S5, Supplementary Material online), certain ligand-binding residues can directly interact with interface residues. We found that mostly these direct binding site—interface interactions are responsible for the stronger correlation (*R*^2^) in MBS homomers: when they are excluded from the analysis, the difference between the MBS and SBS homomers is much smaller, and the effect of binding site type disappears in the partial regression, thus, the difference between MBS and SBS homomers can be explained with the covariates (supplementary fig. S6*G*, Supplementary Material online and supplementary table S7, Supplementary Material online). In the case of MBS homomers with a single multichain-binding ligand (supplementary fig. S7, Supplementary Material online and supplementary table S8, Supplementary Material online), the difference is not significant in any of the comparisons. Thus, the dynamical coupling between the interfaces and ligands of MBS homomers is primarily the consequence of the proximity of MBS ligands to the interface, and is not necessarily related to the topology of their fold (note that unlike for *R*^2^, the effect of such direct interactions on *Z*-score is small).

### Multimerization may not Directly Enhance Function in a High Fraction of SBS Homomers

**Fig. 7.**
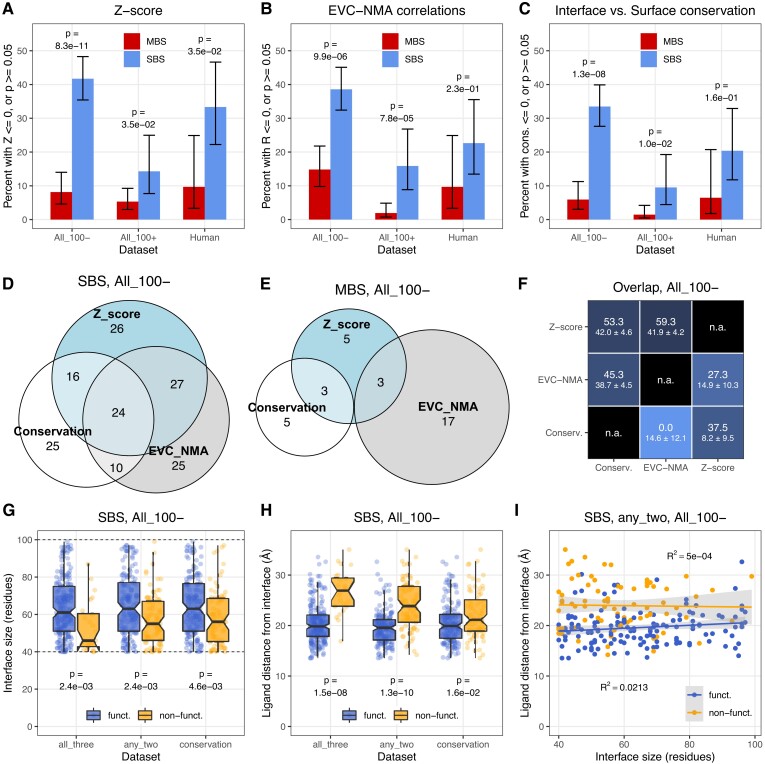
A high fraction of SBS homomers interfaces shows no clear signs of functional coupling with their ligand, information transfer between the subunits, or conservation of interfaces. As MBS homomers have larger interfaces than SBS homomers, complexes with less than 100, and more than 100 interface residues were analyzed separately. (*A*) Sequence coevolution (*Z*-score). The number of proteins where *Z*-score is not positive, or the difference between interface and solvent-accessible surface is not significant is much higher in SBS homomers than in MBS homomers, and reaches 41.9% in structures with interfaces below 100 residues. (*B*) The correlations between sequence coevolution and dynamics indicate that in 38.7% of SBS homomers with interfaces below 100 residues, ligand-interface couplings and NMA dynamics are independent. (*C*) The conservation of proteins indicates that in 33.8% of SBS homomers with small interfaces, the interface is not significantly more conserved than the solvent-accessible surface. Only structures that were also included in figure 4 or 6 were used, thus, *Z*-score or *R*^2^ was determined for them. (*D* and *E*) Venn diagrams of the overlaps between the putatively non-functional sets of the three methods in SBS and MBS homomers. Only complexes with less than 100 interface residues were included. The numbers indicate the absolute number of homodimers in each group. (*F*) The matrix of pairwise overlaps between the three putatively non-functional sets (< 100 interface residues). The upper-left triangle shows SBS homomers, the lower-right triangle MBS homomers. The numbers indicate the overlap in percent, with the random expectation below (±SD). (*G*) The size of the interface is smaller in the putatively non-functional and functional sets of SBS homomers even within the group with less than 100 residues. Three non-functional sets were defined: 1) the intersection of all three methods (*Z*-score + EVC-NMA + conservation); 2) the intersection of any two methods; and 3) as non-conserved interfaces. (*H*) The distance of ligands from the interfaces is larger in the putatively non-functional sets than in the functional sets of SBS homomers. (*I*) Interface size and the distance of ligands from the interface are independent in all cases. The panel shows the set with the strongest correlation (*R*^2^ = 0.0213), the intersection of any two methods.

The results above indicate that multimerization is affected by ligand binding and interface conservation much more in MBS homomers than SBS homomers. However, they do not answer the question in what fraction of homodimers the interface is likely to have no effect on function. We estimated this fraction using the magnitude and significance of interface conservation (fig. 3), *Z*-score (fig. 4) and *R*^2^ values (fig. 6) using the complexes where ligand binding residues coevolve significantly stronger with each other than with surface residues (*P* < 0.05, Wilcoxon tests with Benjamini–Hochberg correction for multiple testing). We assumed that negative or not statistically significant interface conservation, *Z*-score, or *R*^2^ are likely to be present in complexes where the interface does not contribute to function. Since the size of interfaces is variable, influences the power of analysis, and MBS homomers have generally larger interfaces than SBS homomers (Abrusán and Marsh 2019b) (see also fig. 2), we split the full dataset into two groups, with the number of interface residues below 100 and above 100. The human dataset is too small for a similar split. Homomers with two MBS ligands (fig. 7) and only one MBS ligand (supplementary fig. S8, Supplementary Material online) were compared to SBS homomers separately.

Our results show that the fraction of putatively non-functional dimers is significantly higher in SBS homomers than in MBS homomers, both in the full and human datasets (fig. 7*A*–*C*, see also supplementary fig. S8*A*–*C*, Supplementary Material online). In the case of dimers with interfaces below 100 residues, 41.9% of SBS homomers and 8% in MBS homomers show no significantly stronger coevolution of ligands with interfaces than with surfaces (fig. 7*A*). In the case of complexes with larger interfaces, the difference is less pronounced, but still significant. The correlations between evolutionary couplings and NMA dynamics show a comparable pattern (38.7% in SBS homomers, fig. 7*B*), although in MBS complexes with less than 100 interface residues, the fraction of non-significant correlations is higher, 15% (fig. 7*B*). The conservation of interfaces shows a similar albeit somewhat weaker pattern (fig. 7*C*): 33.8% of SBS homomers with small interfaces have interfaces that are not more conserved than the solvent-accessible surface, while only 6% of MBS homomers. However, in MBS homomers, this is likely to be an underestimate, because a significantly higher fraction of their interface residues is affected by ligand binding and long range effects of binding sites on conservation (Jack et al. 2016) than in SBS homomers, and an unambiguous separation of dimer functionality and multichain-binding site conservation is probably not possible. The smaller human dataset shows a similar trend as the full dataset, although the difference is significant only in the case of *Z*-scores (fig. 7*A*). In homomers with a single MBS ligand (supplementary fig. S8, Supplementary Material online), the fraction of complexes identified as putatively non-functional is higher than in complexes with two MBS ligands (supplementary fig. S8*A*–*C*, Supplementary Material online), particularly in the dataset based on interface conservation. This might result from some structures having misplaced ligands, which do not bind a binding site; however, the small number of these homomers (supplementary fig. S8*E*, Supplementary Material online) results in low power to detect significance, an in considerable uncertainty in the pattern.

Finally, using the structures of the full dataset with less than 100 interface residues, we examined to what degree the lists of putative non-functional complexes identified by the three methods overlap (fig. 7*D*–*F*, see also supplementary fig. S8*D*–*F*, Supplementary Material online). Each of the three methods is likely to be influenced by stochasticity, especially in proteins where the effect size is small, and they are also based on different assumptions. When using conservation, we assume that only conserved interfaces are functional, even though constructive neutral evolution can result in conserved but non-functional traits (Stoltzfus 1999; Muñoz-Gómez et al. 2021); *Z*-score assumes that function is primarily related to ligand binding (it is agnostic about its nature, though); while couplings-NMA *R*^2^ assumes that function is related to the dynamics of the complex. In addition, residue coevolution is based on larger alignments than conservation, therefore *Z* and *R*^2^ might be less sensitive than conservation. We found that in SBS homomers, there is a considerable overlap (45–59%) between any pair of the three sets (fig. 7*D* and *F*, upper triangle). However, due to the large percentage of putatively non-functional complexes in SBS homomers, the random expectation for the overlaps is also high, 39–42% (fig. 7*F*, upper triangle). The fraction of SBS homomers identified as non-functional by all three methods is 10.8% (24/222; rand. exp.: 5.5 ± 1.3%), while the fraction identified by at least two methods is 34.7% (77/222; rand. exp.: 32.5 ± 1.7%).

In the case of MBS homomers, the overlap between the groups is much smaller, 4.4% (6/135, fig. 7*E*). However, at least for the ones with two MBS ligands, the observed overlap with *Z* distance is still higher than the random expectation (2.4 ± 1.1%), even though the absolute number of putatively non-functional complexes is low in all three groups (fig. 7*E* and *F*, lower triangle).

### Effects of Long-range Binding-site Constraints on Function

Recently it has been demonstrated that conserved catalytic/binding sites induce long-range evolutionary constraints within proteins, which can have measurable effects on residue conservation even more than 20 Å from the binding site (Jack et al. 2016), although the range is highly variable between proteins (Echave 2021). We hypothesized that besides interface size, this effect—the distance of the ligand from an interface—is likely to be one of the factors that determine whether an interface contributes to the biochemical function of a complex in SBS homomers. Using dimers with <100 residues in the interface, we examined whether, in putatively non-functional SBS homomers, the binding sites are located further from the interface than in functional ones. We defined three putatively non-functional sets: the intersection of all three methods (*Z*-score + EVcouplings-NMA correlations + conservation), the intersection of any of the two methods, and non-conserved interfaces (see fig. 7*D*). We found that even below 100 residues the size of the interface has an effect in all three cases (fig. 7*G*). In the putatively non-functional complexes, ligands are located further away from the interface than in the functional complexes (fig. 7*H*), especially in the intersections of all three, and any two methods, where in most putatively non-functional dimers the ligand is located further than 20 Å from the interface. The effect is weakest in the conservation set, where ligand-interface coevolution was not used to infer functionality (fig. 7*H*). Interface size and ligand distance are uncorrelated (with the highest coefficients of determination [*R*^2^] being 0.0213, see fig. 7*I*), indicating that the two effects are independent. These results indicate that long-range evolutionary constraints of binding sites are relevant for complex functionality, however, the effect of interface size is stronger, and it depends less on the method used to determine the non-functional set.

### MBS Homomers are Less Buffered in the Human Metabolic Networks than SBS Homomers

**Fig. 8.**
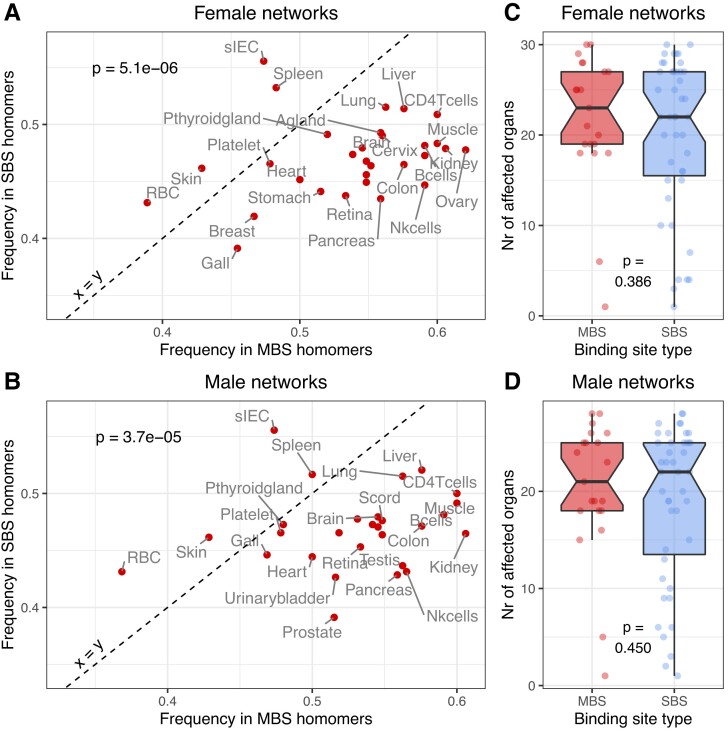
MBS and SBS homomers have a different effect on metabolism. (*A* and *B*) The fraction of MBS and SBS homomers where the flux is significantly affected by a knockout in every organ specific network. Knockouts of MBS homomers are significantly more likely to result in changes in flux than knockouts of SBS homomers both in male and female networks, suggesting that the activity of MBS homomers needs to be regulated more tightly than the activity of SBS homomers (see also supplementary fig. S9, Supplementary Material online). However, we found no differences in the number of organs that are affected by the knockout of an MBS or SBS protein (*C* and *D*).

Finally, we examined whether the structural differences between the two complex types also result in functional differences. Most proteins in the human dataset have enzymatic activity, thus, we tested whether they have different importance in the human metabolic network. We used organ-specific, genome-scale networks extracted from the female and male whole-body human metabolic networks (Thiele et al. 2020). In addition to dimers, we also used homomers with higher unit numbers (selected using the same criteria as dimers) to increase the number of proteins in the analysis (supplementary table S9, Supplementary Material online). For every human homomer from our dataset present in the metabolic network (altogether 165 homomers, of which 112 are dimers with 40+ interface residues), we simulated their knockout in every organ-specific network, and performed flux variability analysis (FVA) before and after the knockout (see Methods). FVA quantifies the feasible range of metabolic flux values (i.e., the rate at which substrates are converted to products through metabolic reactions) and enables quantifying how much each knockout affected the metabolic network. We assumed that a reaction was significantly affected by a knockout if its flux was reduced by more than 50%. We found that in most organs, the knockout of MBS homomers results in one or more reactions with a significantly reduced flux in 45–65% of MBS homomers, but only in 40–50% of SBS homomers (fig. 8*A* and *B*; supplementary fig. S9*A*and*B*, Supplementary Material online). The pattern is not influenced dramatically by using only homodimers with 40+ residues in their interfaces (112 proteins, fig. 8), or using the extended set of human homomers, including those with higher unit numbers (165 proteins, supplementary fig. S9, Supplementary Material online); in the latter, the trend is even more pronounced. However, we found no clear differences in the numbers of affected reactions per gene or the number organs of affected by an MBS or SBS knockout (fig. 8*C* and *D*, supplementary fig. S9*C*and*D*, Supplementary Material online). Although the human dataset is small, taken together, these results suggest that MBS homomers are potentially regulated differently from SBS homomers, as changes in their activity are more likely to have consequences for metabolism. Based on our previous findings, which show that allostery is more common in MBS than in SBS complexes (Abrusán and Marsh 2019b), and our results above, we speculate that allostery might provide one such level of enhanced regulation.

## Discussion

Generally, our analysis indicates that multimerization is unlikely to contribute to the biochemical function in a significant fraction of homomers, particularly with single-chain binding sites. However, it also shows that in most dimers, even though a neutral hydrophobic ratchet does shape the evolution of interfaces, interface residues nevertheless coevolve significantly more with each other than with non-interface residues (fig. 2*A*–*G*), most likely to ensure correct assembly and minimize the number of solvent-exposed hydrophobic residues. Unlike coevolution across interfaces, interface conservation scales significantly with interface size also above the 40-residue threshold (fig. 3*A*–*E*), but when the full 40+ dataset is analyzed, the majority of interfaces are conserved to some degree (74.3% in SBS and 93.4% of MBS homomers, fig. 3*G*).

The effect of the hydrophobic ratchet is visible in the correlation between the amino acid frequencies in interfaces and gnomAD missense variants (fig. 2 and supplementary fig. S2, Supplementary Material online). However, the two are not in equilibrium, neither in MBS nor in SBS homomers, and certain hydrophobic residues like cysteine are produced at much higher frequencies by mutations than their frequency in the interfaces. This suggests strong purifying selection against off-target disulphide bridges (figs. 2*H*–*L* and 3*D*–*E*), consistent with their high enrichment in pathogenic mutations (Iqbal et al. 2020) and the general depletion of cysteines in protein surfaces due to their high reactivity (Marino and Gladyshev 2010). The results also show that the magnitude of coevolution across interfaces, and conservation has only a modest (albeit significant) effect on their hydrophobicity (figs. 2*G* and 3*I*). Thus, hydrophobicity of these interfaces scales little with the strength of selection, and it is primarily the result of a neutral process, which is in agreement with the predictions of the hydrophobic ratchet. However, this also means that the ratchet shapes also the hydrophobicity of functional interfaces, and the level of hydrophobicity predicts poorly whether an interface is functional or not.

The coevolution of ligands with interfaces shows a clear difference between the two complex types, ligands of SBS homomers coevolve much less with interfaces than in MBS homomers (fig. 4 and supplementary fig. S3, Supplementary Material online). The coevolution of interface residues with ligands is also correlated with their dynamics, and the ability to transmit information from ligands through the interface is much more pronounced in MBS homomers than in SBS homomers (fig. 6 and supplementary fig. S5, Supplementary Material online). While this finding demonstrates only a capacity and does not prove that there is actual, biologically relevant communication between the subunits, it helps to explain previous findings by us (Abrusán and Marsh 2019b) and subsequently by others (Xie and Lai 2020) (see also (Changeux 2013)), that complexes with multichain binding ligands are much more likely to be allosteric than with single-chain binding ligands. The results also indicate that one of the most important factors enabling such communication is simply the closeness of ligand-binding residues to interface residues, which is in agreement with the recent finding that the fold composition of known allosteric proteins is not dramatically different from other proteins (Xie and Lai 2020), and fold-composition may not be a major factor in determining whether a protein is allosteric or not. The possibility that MBS homomers are regulated differently than SBS homomers (e.g., by allostery) is indirectly supported by the finding that knockouts of MBS homomers in metabolic networks are more likely to result in reduced flux and blocked reactions (fig. 8).

We estimated the fraction of complexes that are likely to be non-functional with three different methods: coevolution between the binding sites and interface (*Z*-score), the correlation between motions and coevolution (*R*^2^), and with the fraction of complexes with non-conserved interfaces (fig. 7 and supplementary fig. S8, Supplementary Material online). The first two assume that functionality is related to ligand binding (i.e., biochemical function and dynamics), while conservation is agnostic about the mechanism of selection, and takes into account that it may operate at a different level. For example, it has been demonstrated that multimerization affects the degradation rate of proteins (Mallik and Kundu 2018; Abrusán and Marsh 2019a), thus, it may affect the lifespan of proteins in the cell rather than biochemical function. In addition, recent work has demonstrated that mutations in protein surface can result in polymerization (Garcia-Seisdedos et al. 2017) and changes in their subcellular localization (Garcia-Seisdedos et al. 2022), suggesting that multimerization may also affect their cellular distribution. We focused on dimers, but in the case of larger, cyclic homomers with multiple subunits like ion channels or fibrils, complex formation might be adaptive by minimizing the amount of information that is necessary to encode a given molecular machine in the genome (Johnston et al. 2022).

The three different methods show a broadly similar pattern: the vast majority (85–95%) of MBS homomers is predicted to be functional, even when their interfaces are relatively small, or bind a single MBS ligand (figs. 7*A*–*C* and 8*A*–*C*). In contrast, in SBS complexes functionality depends largely on the size of the interface, and in small to moderately-sized interfaces the three methods predict 34–42% of the dimers as putatively non-functional (figs. 7*A*–*C* and 8*A*–*C*).

The comparison of the putatively non-functional sets identified by the three methods in dimers with small/medium-sized interfaces (less than 100 residues) shows that in MBS homomers, there is little overlap between them (4.4%, 6 dimers), while in SBS homomers they overlap considerably, nevertheless, the three sets are not identical (fig. 7*D*–*E*, supplementary fig. S8*D*–*E*, Supplementary Material online). This is likely to reflect the fact that they quantify different aspects of functionality, but also the consequence of stochasticity. One way of reducing the effect of stochasticity is to use the intersections of the different methods. The intersection of all three sets identifies 10.8% of SBS homomers as non-functional; while intersection of any two methods identifies 34.7% of SBS dimers with small interfaces as non-functional. However, many dimers are unlikely to be functional even in the presence of a conserved interface, in fact, purifying selection might be necessary to maintain non-functional interfaces (Hochberg et al. 2020), and the characteristics of folds of SBS homomers are frequently comparable to monomers (Abrusán and Marsh 2019a), thus this might still be an underestimate. The maximum number of gratuitous complexes might be estimated with the *Z*-score (41.9%, fig. 7*A*), or even with the union of *Z*-score and conservation sets (57.6%, fig. 7*D*), because having conserved interfaces or positive *R*^2^ do not automatically mean that multimerization is functional. While these fractions are high, they seem to agree with a recent study (Marciano et al. 2022), showing that many homomers have several oligomeric forms in solution, and for half of the 17 proteins analyzed by these authors the available structures in the PDB underestimate their real structural diversity; thus, their quaternary structure may not influence function, or their PDB entry may not be the functional or even the dominant form.

So far, work on protein-complex neutrality focused on homomers, however, several findings in heteromers are also consistent with the idea that some of their subunits may not contribute to function, and redundant subunits might be common in them. Different subunits of the same complexes frequently evolve very differently (Matalon et al. 2014), their subunit composition can vary between species (Fokkens and Snel 2009; Seidl and Schultz 2009), and even between different cell types of the same species (Ori et al. 2016). A recent study that examined coevolution in protein–protein interactions at a genomic scale in bacteria (Cong et al. 2019) found that coevolution across interfaces is strongest in binary complexes, and it substantially decreases with the number of subunits, also suggesting that larger complexes have functionally redundant components. Together with the work on homomers, these findings suggest that the acquisition of neutral subunits is common in protein complexes.

## Materials and Methods

### Selection of Proteins and Structures for Analysis

We have compiled two lists of homodimers: one that is based on all proteins and species of the PDB, excluding viruses (“All”), and one having only human proteins (“Human”). In the first (“All”) dataset, we selected the PDB entries that cover at least 90% of their UniProt sequence (using the SIFTS database (Dana et al. 2019)), have a biologically relevant ligand that is not a metal (as defined by the BioLiP database (Yang et al. 2013)), and their resolution is better than 3 Å. A minimum length of 100 residues was required for the UniProt sequence. We used the first biounit of every PDB entry and excluded PDB entries where the biounit is a multiplication of the asymmetric unit, that is, if the asymmetric unit is a monomer while the biounit is a dimer. When a protein had multiple PDB entries, we used only the structures with the highest number of chains, and in the case of entries with similar topologies, with the largest ligand. We removed redundancies from the selected proteins by clustering them with usearch (Edgar 2010) at 30% sequence similarity cutoff, and kept only the cluster centroids. Since the number of SBS homomers was more than twice as high in the list as the number of MBS homomers, to reduce the computing time in the downstream analyses, we removed half of the SBS homomers, by sorting their PDB ids and keeping only every second id. Finally, from the remaining structures, we removed the ones where the entry has biounits with different numbers of chains, thus, the complex may not be a dimer. For the human dataset, a similar procedure was performed with the following differences: we required a lower 75% coverage of the UniProt sequence in the PDB entries; no redundancy filtering (clustering) was applied, and no SBS homomers were removed.

### Identification of Interface Residues and Interactions

We used the RINerator tool (Doncheva et al. 2011) to identify residue interactions, which rolls a probe with a 0.25 Å radius over the van der Waals surface of each atom to identify residues in contact. Interface residues were defined as residues from different protein chains that form direct interactions. Residues that bind ligands were not considered interface residues unless they also bind to another protein chain. Since the structures are not always symmetrical, and the number of interface residues can be different on the two chains, we used the sum of interface residues on both chains in the downstream analyses as interface size.

### Determining the Type of the Ligand-Binding Site and Ligand Distance from the Interface

The type of the ligand-binding site (i.e., multi-chain vs. single-chain) was determined in two steps. First, as an initial estimate, we used the ligand-binding residues of the BioLiP database to identify binding sites that have residues from a single or multiple protein chains. As BioLiP is based on asymmetric units, while we used the biounits in our analyses, we validated the binding site type in all structures with the AREAIMOL tool of the CCP4 package (Winn et al. 2011). Ligand binding residues in biounits were identified as follows: we calculated the solvent-accessible area of every residue in the biounits with AREAIMOL, with and without ligands. Ligand binding residues were determined as the residues where the solvent-accessible area changes upon ligand binding. We parametrized AREAIMOL to maximize the similarity with the residues identified by BioLiP, which resulted in a probe size of 0.21 Å. These probe sizes resulted in strong correlations with BioLiP residues (*R*^2^ = 0.988, supplementary fig. S10, Supplementary Material online).

The distance of every ligand from the interface was calculated as the average of all possible distances between the residues of the interface, and the residues of the ligand binding site that is closest to any given interface residue.

### Calculation of Evolutionary Couplings

Evolutionary couplings were calculated for the protein sequence of the first chain of every homodimer with the EVcouplings package (Hopf et al. 2019) with the standard protocol (which uses plmc), using their sequence in the PDB structure (i.e., seqres entry). We used the uniref90 sequences as the sequence database, and required 75% minimum sequence coverage, 70% minimum column coverage, 5 jackhmmer iterations with 0.7 domain and sequence threshold, and 100 plmc iterations. In addition, the following parameters were used: clustering threshold (theta) = 0.8; lambda_J = 0.01; lambda_h = 0.01, ignore_gaps = True; lambda_J_times_Lq = True. The raw coupling scores (cn) were used in the downstream analyses, which have a high agreement with the output of other packages, like Gremlin.

In the case of figure 2, the coupling score distributions of interface interactions are skewed, and we used the Yeo–Johnson transformation (a generalization of the Box–Cox transformation, which can also transform negative values) to improve their normality. The lambda parameter was adjusted for every protein separately, to maximize the normality of the interactions between interface and non-interface residues, which was used as the reference distribution (see fig. 1). In the case of other analyses involving ligand-binding residues (e.g., fig. 4), the distributions of coupling scores were much closer to normality and were not transformed. In all analyses that use EVcouplings output, we excluded the proteins where the ratio of the effective number of sequences and sequence length (sites) was less than one.

### Variant Analyses

For every homomer, we identified its corresponding Ensembl protein sequence using release 75 of Ensembl, and the sequences of the PDB structures were aligned and matched with the Ensembl's sequence using MUSCLE v3.8 (Edgar 2004). Only PDB-Ensembl sequence pairs where the sequence similarity is higher than 90% were used. When a protein had multiple isoforms, we selected the most similar one to the UniProt reference. We used the gnomAD database (Karczewski et al. 2020) to identify human missense variants in the regions corresponding to the Ensembl sequences’ protein interfaces, and solvent accessible surface. Due to the relatively small number of variants in the interfaces (1392 in MBS and 1948 in SBS homomers), the allele frequencies of the variants were not used. The following seven amino acids were classified as hydrophobic (Hochberg et al. 2020): Cys (C), Phe (F), Ile (I), Leu (L), Met (M), Val (V), and Tyr (Y). Solvent-accessible surface was defined as residues that are not part of the interface, do not bind ligands, and their relative solvent accessibility (RSA) is larger than 0.2. The RSA of residues was calculated as the ratio of solvent accessible surface in the structure (calculated with DSSP (Touw et al. 2015)) and the solvent-accessible area in a Gly-X-Gly tripeptide (Miller et al. 1987).

### Estimating Conservation

The degree of conservation was estimated using the ConSurf database (Ben Chorin et al. 2020). ConSurf provides normalized conservation scores at the residue level for most structures in the PDB. We downloaded the consurf summary files for every structure we used (note that the PDB is highly redundant, and many entries have identical sequences. In such cases, ConSurf usually has only one entry for all PDB structures with identical sequences, and it may not have the same ID than the one we used in our analysis). Using the summary files, we calculated the average conservation score for the interface residues and the residues of the solvent-accessible surface. The difference between the two averages (surface–interface, which results in positive values when the interface is more conserved than the surface) was used in the downstream analyses.

### NMA and Calculation of Cross-Correlated Motions

From every structure we removed all waters and ligands that are absent in the BioLiP database (Yang et al. 2013), and kept only the ligands that are likely to be biologically relevant. We used the first biounit of every homomer in the calculations. Next, we performed an all-atom NMA on the protein complex with the “aanma” function of the Bio3D R package (Yao et al. 2016; Grant et al. 2021). To reduce computational load and memory requirements, hydrogens were not added, and we enabled the use of rotational-translational blocks. As all-atom NMA is very memory intensive, and depends on the number of atoms, NMA has effectively limited the maximum size of structures to approximately 2000 residues. Finally, the normal modes were used to calculate the dynamic cross-correlation matrix with the dccm function of Bio3D. All modes were used.

### Flux Variability Analysis

We reconstructed organ-specific subnetworks starting from the Harvey/Harvetta v1_03c human whole-body genome-scale metabolic network (Thiele et al. 2020). First, the male (“Harvey”) and female (“Harvetta”) networks were converted from Matlab to the SBML format, and for each organ, organ-specific reactions and relevant exchange reactions were extracted. Then, metabolites in blood were made boundary conditions (i.e., assumed constant) allowing each organ subnetwork to function independently. In total, 28 male, and 30 female organ-specific networks were obtained. FVA was used to compute “wild type” flux ranges (i.e., the minimum and maximum flux values allowed for each reaction) in each network. To estimate the importance of individual homomers in metabolism, we simulated a knockout for each human homomer gene present in the networks (altogether 165) in every male and female organ-specific network. Subsequently, FVA was rerun, and the effect of the knockout was measured by the change in fluxes in the knockout network compared to the wild type. Reactions with absolute flux values below 10^−6^ mol/day were considered blocked.

### Data Processing, Statistics, and Visualization

Data processing was done with in-house Perl scripts. Statistical analyses were performed with R v3.6, and v4.1.2. Type III ANCOVA was performed with the sasLM R package, and Yeo–Johnson transforms with the VGAM R package. Graphs were plotted with the ggplot2 and cowplot R packages. Protein structures were visualized with UCSF Chimera and PyMol. Processing of genome-scale metabolic models, knockouts, and flux variability analyses were performed with the COBRApy python package (v0.9.1) (Ebrahim et al. 2013), using the IBM CPLEX 12.8 solver.

## Supplementary Material

Supplementary data are available at Molecular Biology and Evolution online.

## Supplementary Material

msad070_Supplementary_Data

## Data Availability

Supplementary data and code to reproduce the figures and supplementary figures are available at Zenodo, https://doi.org/10.5281/zenodo.7784018.
