# Supplementary material for: An Assessment of Quaternary Structure Functionality in Homomer Protein Complexes
Source: Mol Biol Evol. 2023 Mar 22;40(4):msad070. doi: 10.1093/molbev/msad070 (PMC10118308; doi:10.1093/molbev/msad070)
Supplement: msad070_Supplementary_Data [file msad070_supplementary_data.zip › SFigures.pdf]

SUPPLEMENTARY FIGURES
to
"An assessment of quaternary structure functionality in homomer protein complexes"

by György Abrusán and Carles Foguet

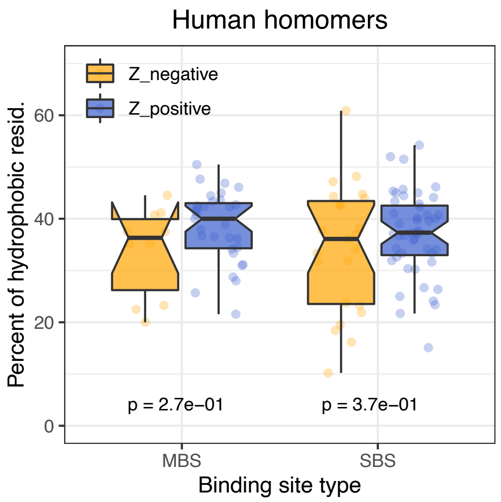

Figure S1. In the human dataset, the fraction of hydrophobic residues in the interfaces of complexes with negative Z-score is not significantly different from those with positive Z-score.

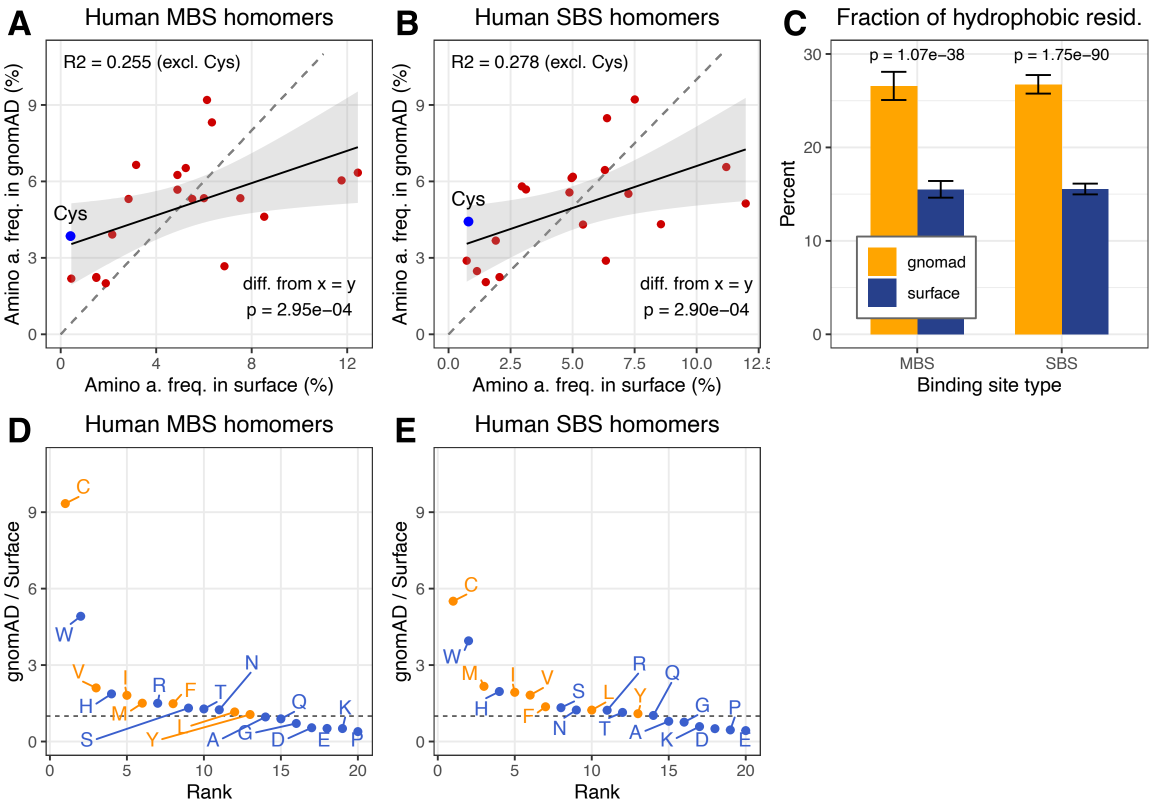

Figure S2. (see legend on next page)

Figure S2. The frequencies of mutations and amino acids in solvent accessible surfaces. **(A and B)** The frequency of specific amino acids in the solvent accessible surface and surface missense mutations (excluding Cysteine) shows a correlation where the slope is significantly different from one in both homomer types (F statistic). **(C)** The frequency of hydrophobic amino acids is much higher in surface missense mutations than in the surface itself (tests of proportions). **(D and E)** The frequency of cysteines in missense mutations is 6 to 9-fold times higher than in the surface, indicating strong selection against cysteines. Besides cysteine, tryptophan (W) is also 5-fold enriched in missense mutations, mostly due to converting the CGG, AGG arginine codons to tryptophan (TGG).

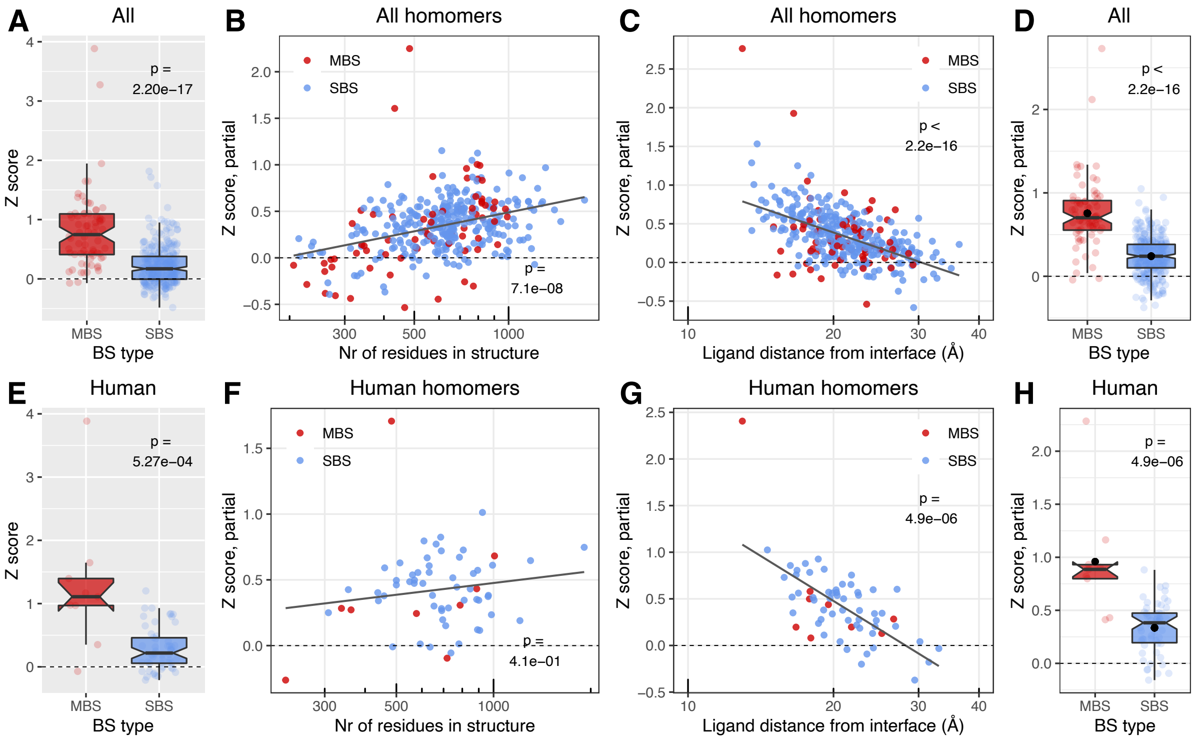

Figure S3. MBS homomers with a single multichain binding ligand show similarly strong coevolution with ligands as homomers with two multichain ligands. **(A)** The raw Z scores of MBS and SBS homodimers in the full dataset. **(B, C and D)** ANCOVA partial regressions between Z score and the size of the structure (B), the distance of ligands from the interface (C), and binding site type (D). The black dots of panel G indicate the means. See the full ANCOVA results in Table S4. **(E, F, G and H)** The human dataset shows similar trends as the full dataset. (See also Table S4)

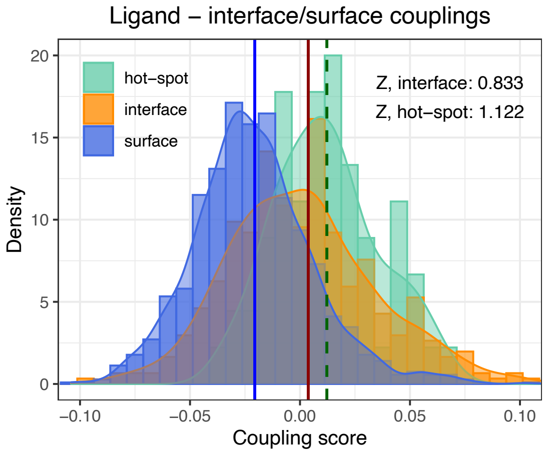

Figure S4. Distributions of coupling scores in E. coli Thymidylate synthase. Hot-spot residues, which are the most responsible for the dynamic coupling between the two subunits of the dimer (and inter-ligand communication) show stronger coevolution with ligand-binding residues than the interface. Z was calculated similarly to Figure 4, as the difference of the hot-spot and interface means from the mean of the solvent-accessible surface distribution, divided by its SD.

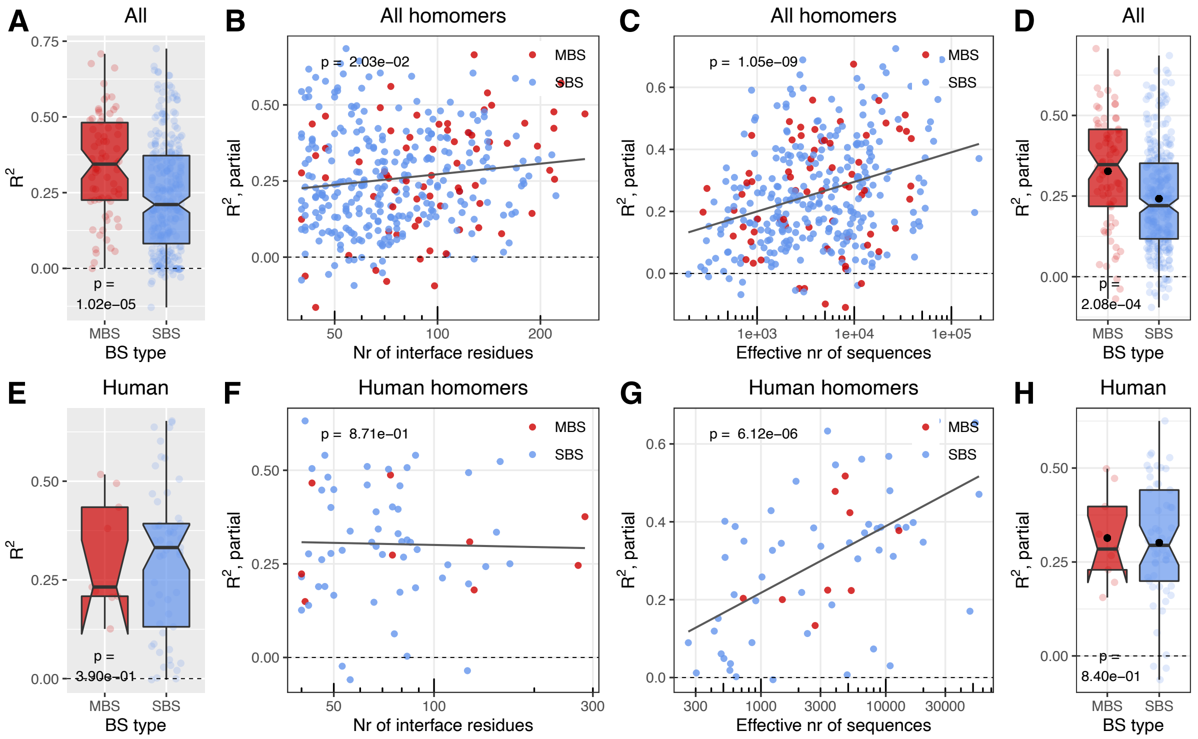

Figure S5. MBS homomers with a single multichain binding ligand show similar correlations between dynamics and coevolution as MBS homomers with two ligands. **A)** Raw R^2 values of the full nonredundant dataset. **B-D)** Partial correlations of the full dataset (see Table S6 for full ANOVA results.) **E-H)** In the small human MBS dataset (8 proteins), we found no significant difference between the MBS and SBS homomers (see also Table S6).

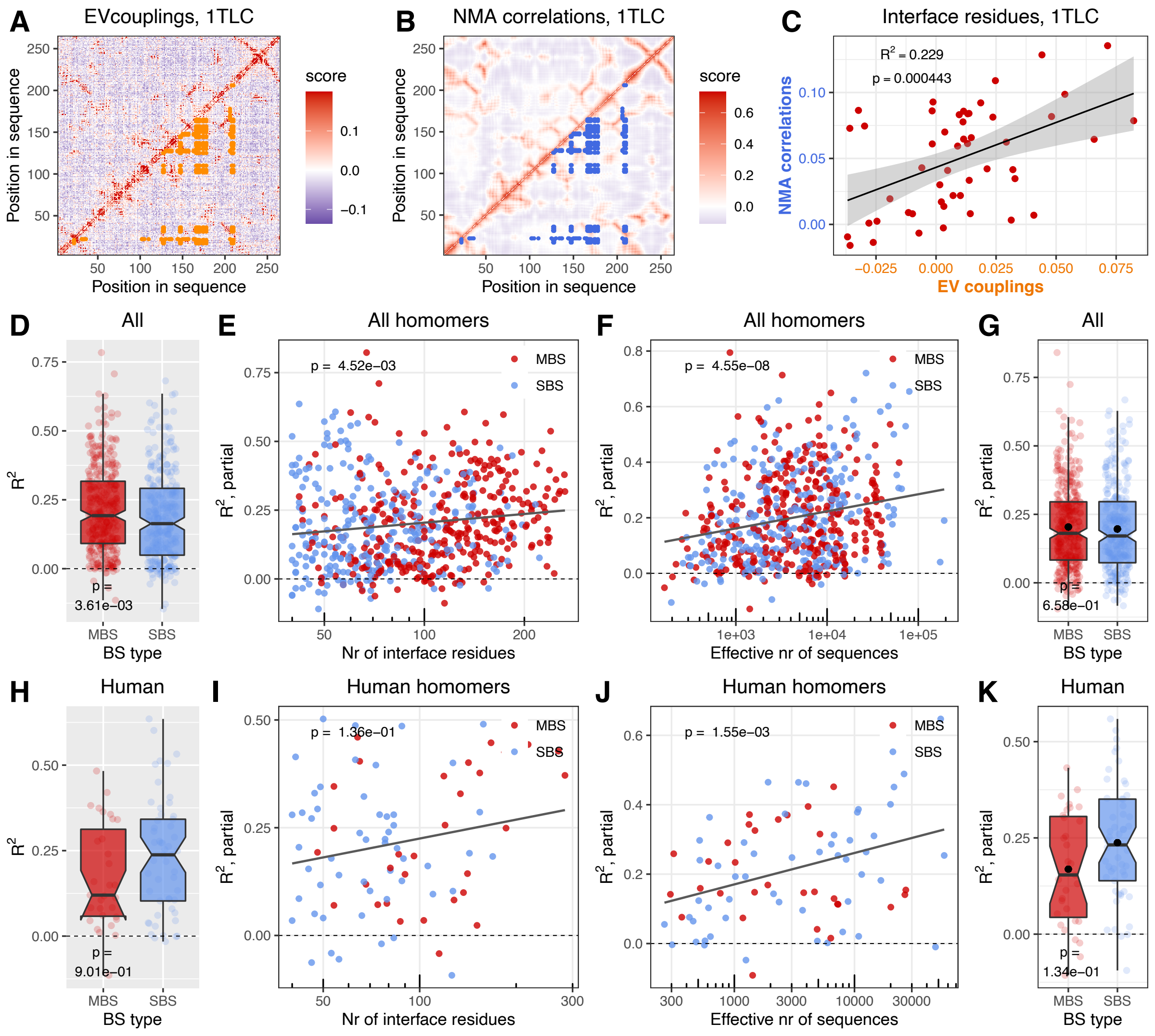

Figure S6. Excluding ligand-binding and interface residues that are directly in contact in the structures significantly reduces the difference between MBS and SBS homomers. **(A)** Matrix of evolutionary coupling scores of E. coli Thymidylate synthase, orange marks represent couplings between ligand-binding and interface residues. **(B)** Matrix of cross-correlated motions in chain A of the Thymidylate synthase structure (PDB id: 1TLC). Blue points represent the correlated motions between ligand-binding and interface residues. **(C)** Correlation between (A) and (B); each point represents an interface residue, for which the average coupling score and average dynamic correlation was calculated with the ligand-binding residues. **(D)** Raw R^2 values of the two complex types. **(E-F)** Partial correlations. (See also Table S7). Note that the partial correlation of the binding site type (G) is not significant. **(H-K)** In the human dataset, we found no significant difference between MBS and SBS homomers (See also Table S7).

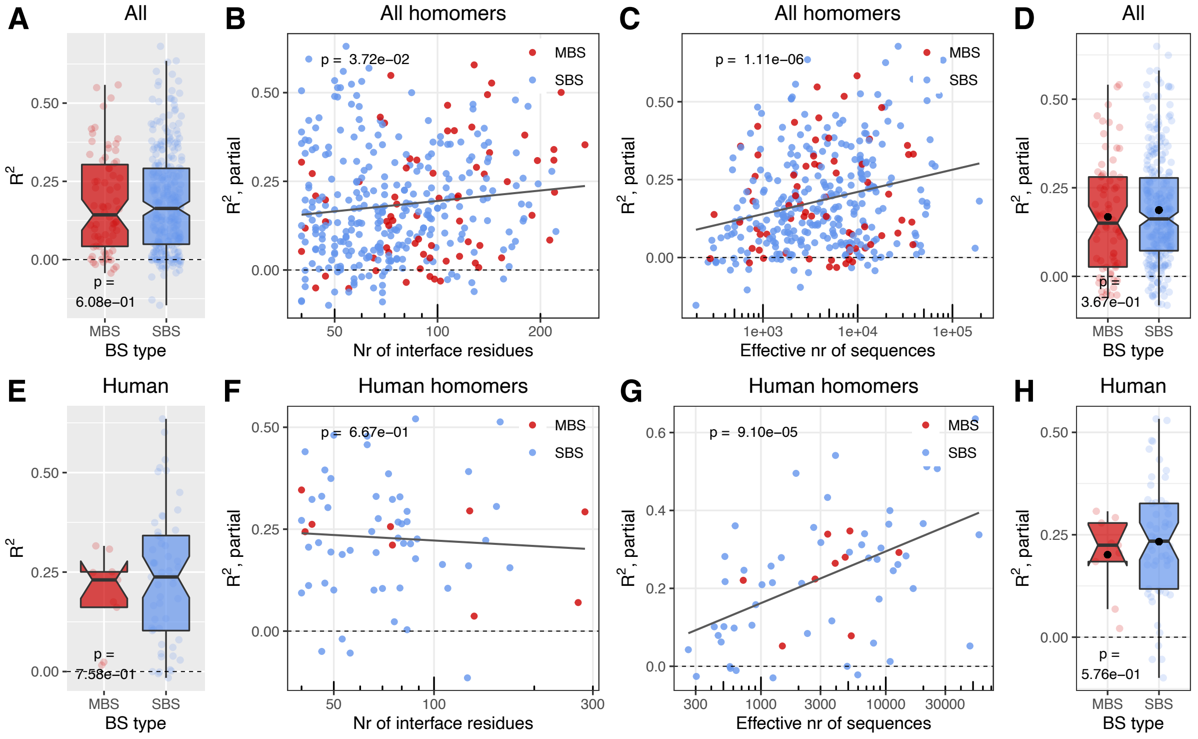

Figure S7. The same as Figure S6, but using MBS homomers with a single multichain binding ligand. **(A-D)** The full, nonredundant dataset. **(E-F)** The human dataset. See also Table S8.

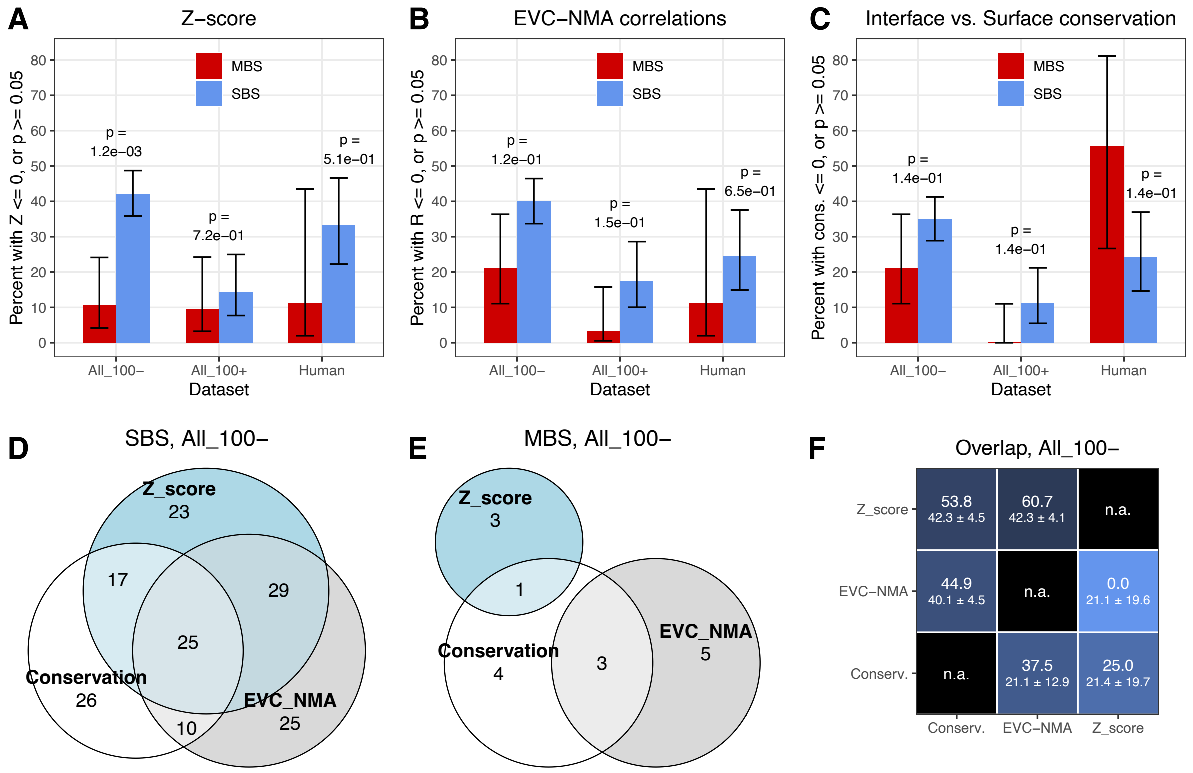

Figure S8. Comparison of homomers with a single MBS ligand with homomers with two SBS ligands. **(A)** Coevolution between the interface and ligand binding residues (Z-score). **(B)** The correlation between sequence coevolution and dynamics. **(C)** Conservation based on ConSurf. The difference between MBS and SBS homomers is less pronounced on all panels, and most of the comparisons are not significant due the to the small number (and low power) of MBS homomers, although with Z-score and R^2 the same trend is present as on Figure 7. **(D-E)** Venn diagrams of the overlaps between the putatively non-functional sets identified by the three methods in SBS and MBS homomers. Only complexes with less than 100 interface residues were included. The numbers indicate the absolute number of homodimers in each group. **(E)** The matrix of pairwise overlaps between the three putatively non-functional sets (100- interface residues). The upper-left triangle shows SBS homomers, the lower-right triangle MBS homomers. The numbers indicate the overlap in percent, and the random expectation (+/- SD). Note that the values of SBS homomers are slightly different from Figure 7, due to differences in the correction of p-values for multiple testing (Benjamini-Hochberg), which in this analysis included a different set of MBS homomers.

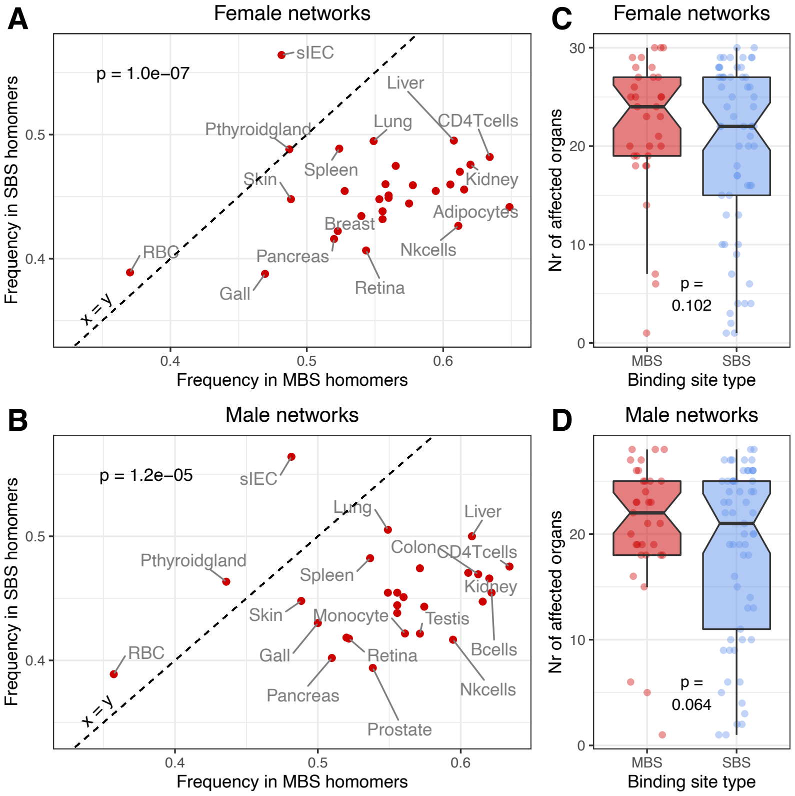

Figure S9. When an extended set of homomers is used in the knockout analysis, which also includes homomers with a higher number of subunits and small interfaces, the differences between the knockouts of MBS and SBS homomers are even more pronounced **(A and B)**, but the difference between the number of affected organs remains not significant **(C and D)**.

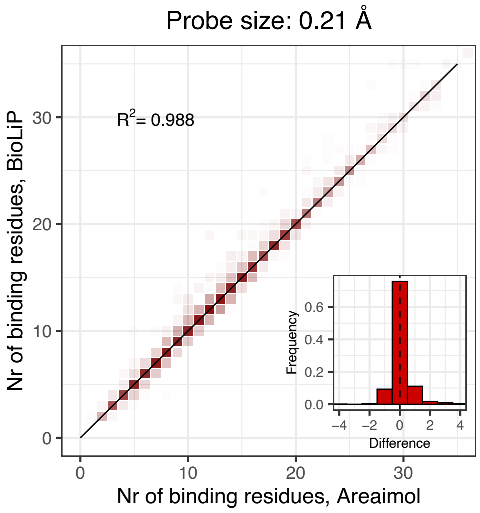

Figure S10. Correlation between the number of residues bound by every small molecule ligand, using the binding residues of the BioLiP database, and the residues identified by AREAIMOL.
